# Global Gene Expression Characterization of Circulating Tumor Cells in Metastasic Castration-Resistant Prostate Cancer Patients

**DOI:** 10.3390/jcm9072066

**Published:** 2020-07-01

**Authors:** Luis León-Mateos, Alicia Abalo, Helena Casas, Urbano Anido, Óscar Rapado-González, María Vieito, Mercedes Suárez-Cunqueiro, Antonio Gómez-Tato, Miguel Abal, Rafael López-López, Laura Muinelo-Romay

**Affiliations:** 1 Translational Medical Oncology (Oncomet), Health Research Institute of Santiago (IDIS), Complexo Hospitalario Universitario de Santiago de Compostela (SERGAS), 15706 Santiago de Compostela, Spain; Luis.Angel.Leon.Mateos@sergas.es (L.L.-M.); urbanoanido@gmail.com (U.A.); mariamercedes.suarez@usc.es (M.S.-C.); miguel.abal.posada@sergas.es (M.A.); 2 Instituto de Salud Carlos III, Centro de Investigación Biomédica en Red de Cáncer (CIBERONC), 28029 Madrid, Spain; oscar.rapado@rai.usc.es; 3 Liquid Biopsy Analysis Unit, Translational Medical Oncology (Oncomet), Health Research Institute of Santiago (IDIS), 15706 Santiago de Compostela, Spain; alicia.abalo.pineiro@sergas.es (A.A.); henacasas@gmail.com (H.C.); 4 Department of Surgery and Medical Surgical Specialties, Medicine and Dentistry School, Universidade de Santiago de Compostela, 15782 Santiago de Compostela, Spain; 5 Vall d’Hebron Institute of Oncology (VHIO), Vall d’Hebron University Hospital, 08035 Barcelona, Spain; mariavieito@gmail.com; 6 School of Mathematics, University of Santiago de Compostela (Campus Vida), 15782 Santiago de Compostela, Spain; agtato@me.com

**Keywords:** circulating tumor cells (CTCs), castration-resistant prostate cancer (CRPC), expression arrays, tumor markers

## Abstract

Background: Current therapeutic options in the course of metastatic castration-resistant prostate cancers (mCRPC) reinforce the need for reliable tools to characterize the tumor in a dynamic way. Circulating tumor cells (CTCs) have emerged as a viable solution to the problem, whereby patients with a variety of solid tumors, including PC, often do not have recent tumor tissue available for analysis. The biomarker characterization in CTCs could provide insights into the current state of the disease and an overall picture of the intra-tumor heterogeneity. Methods: in the present study, we applied a global gene expression characterization of the CTC population from mCRPC (*n* = 9), with the goal to better understand the biology of these cells and identify the relevant molecules favoring this tumor progression. Results: This analysis allowed the identification of 50 genes specifically expressed in CTCs from patients. Six of these markers (*HOXB13*, *QKI*, *MAOA*, *MOSPD1*, *SDK1*, and *FGD4*), were validated in a cohort of 28 mCRPC, showing clinical interest for the management of these patients. Of note, the activity of this CTC signature was related to the regulation of MYC, a gene strongly implicated in the biology of mCRPC. Conclusions: Overall, our results represent new evidence on the great value of CTCs as a non-invasive biopsy to characterize PC.

## 1. Introduction

Prostate cancer (PC) is the most common male malignancy in the Western world. Local treatment with radiotherapy or surgery achieves a high cure rate, but patients with metastatic disease have a poor prognosis, with a 5-year survival rate of less than 30% [1]. Androgen suppression is the standard of treatment for locally advanced and metastatic tumors, but despite the high number of initial responses, most men have progressive disease, which is called castration-resistant prostate cancer (CRPC) [2].

The treatment landscape for patients with advanced or metastatic PC has changed dramatically in recent years, and advances in systemic chemotherapy, new hormonal agents, and the use of Radium 223 or Sipuleucel have significantly improved the overall survival (OS) [3]. However, this increase in therapeutic options has not been accompanied by the development of biomarkers to select the most effective and less toxic treatment for each patient. The key elements determining the prognosis and the decision of when to start or finish treatment in metastatic castration-resistant prostate cancers (mCRPC) patients are the clinicopathological features, serum PSA, and radiological evaluation [2]. However, this approach is not enough to have an accurate evaluation of the disease prognosis and evolution. In addition, nowadays we know that PC is a dynamic disease, with different clones of tumors arising over time in response to different lines of therapy [4].

The study of circulating biomarkers, including circulating DNA and circulating tumor cells (CTCs), has generated major interest because the results may provide prognostic, predictive, response, and even surrogacy information. A considerable number of technologies have been developed to isolate, quantify, and characterize CTCs in recent years, but only the CellSearch platform has been cleared by the FDA for clinical use in metastatic breast, colorectal, and prostate cancers [5]. Several studies have established the prognostic value of CTCs counts for OS in patients with PC [6,7,8,9]. Thus, the presence of ≥ 5 CTCs prior to the initiation of the chemotherapy regimen was associated with a lower OS. Besides this, a decrease in the CTC count below five CTCs has been also associated with a higher OS, as CTC enumeration was investigated as a surrogate end-point for OS in different clinical trials [9,10].

Beyond the enumeration of CTCs or the quantification of circulating DNA, the interest in precision oncology is directed at understanding the molecular pathways involved in tumor development and the mechanisms of resistance to treatment. Thus, in recent years the influence of the alteration of the repair mechanisms of DNA tumors (*BRCA1*, *BRCA2*, *ATM*) and the presence of mutations, amplifications, or splice variants of androgen receptor (AR) have been studied to demonstrate the CTCs’ value as a useful liquid biopsy [11,12,13]. In a previous study, our group addressed the CTC characterization in mCRPC by RT-qPCR and found a gene expression signature composed of *AR*, *CYP19*, *BIRC5*, *TUB1A*, *GDF15*, *RAB7*, and *SPINK1*, with the ability to predict the survival of the mCRPC patients, even improving the enumeration obtained with the CellSearch System [14].

In the present study, we applied a more comprehensive characterization of the CTC population from mCRPC through a global gene expression approach, with the goal to better understand the biology of these cells and identify relevant molecules favoring this tumor progression. This strategy allowed the identification of new CTC biomarkers (*HOXB13*, *QKI*, *MAOA*, *MOSPD1*, *SDK1,* and *FGD4),* with clinical interest in the management of these patients. Our results represent new evidence of the great value of CTCs as a non-invasive biopsy to characterize PC.

## 2. Experimental Section

### 2.1. Patients

A total of 28 mCRPC patients and 15 healthy individuals were prospectively enrolled at Complexo Hospitalario Universitario de Santiago, Santiago de Compostela (Spain). The participants were informed and signed consent was given before their inclusion in the study, according to the Galician Ethical Committee (code of approval: 2011/408). All the individuals in the PC group had a histologically confirmed diagnostic of adenocarcinoma, evidence of progression despite castrate levels of testosterone, and had at least one hormonal manipulation fail, being eligible for systemic chemotherapy based on Docetaxel. Other inclusion criteria were an Eastern Cooperative Oncology Group (ECOG) performance status not greater than 2 and an estimated overall survival (OS) higher than 3 months. Detailed information about patients included in the study is available in (Table 1, Supplementary Table S1). The control group included healthy volunteers with a similar age range and no previous cancer episodes.

### 2.2. CTC Isolation

A gene expression analysis was carried out on blood samples extracted from 9 patients before starting chemotherapy. In parallel, the same protocol was applied to blood samples from 6 healthy donors, establishing the baseline of background from unspecific immunoisolation. The CTC isolation was performed according to the manufacturer’s instructions with the CELLection^TM^ Epithelial Enrich system (Invitrogen, Dynal, Oslo, Norway), which contains beads coated with EpCAM antibodies. Briefly, 7.5 mL of blood was incubated for 30 min at 4 °C with 100 mL of magnetic beads. After washing, CTCs coupled with the magnetic beads were directly resuspended in 100 mL of RNAlater solution (Invitrogen, Carlsbad, CA, USA) and stored at −80 °C until processed for RNA extraction.

### 2.3. Global Gene Expression Analysis

A global gene expression approach was applied to the CTC-enriched fraction from 9 patients and 6 healthy volunteers (Supplementary Table S1). A total RNA extraction, complete Whole Transcriptome Amplification (WTA2, Sigma Aldrich, St. Louis, MO, USA), and gene expression array were performed as described [15,16]. The total RNA was extracted with the QIAmp viral RNA mini kit (Qiagen, Valencia, CA, USA) specifically designed for very low cellularity samples. Subsequent pure RNA was then subjected to a Complete Whole Transcriptome Amplification PCR for 20 cycles using the maximum amount of RNA and Cy3 labeling and hybridization onto Agilent 4x44k gene expression arrays. Upon hybridization, the signal was captured and processed using an Agilent scanner (G2565B, Agilent Technologies, Santa Clara, CA, USA). The scanner images were segmented by the Agilent Feature Extraction Software (v9.5) with the protocol GE1-v5_95 (Agilent Technologies, Santa Clara, CA, USA).

An extended dynamic range implemented in the Agilent software was applied to avoid saturation in the highest intensity range. The Agilent feature extraction was used as raw data for further pre-processing. The processed signal (gProcessed-Signal) value was chosen for the statistical analysis instead of the signal with a substracted background (gBGSubSignal) since it produces a lower average coefficient of variation (CV) in Spike-In and gene replicates [17,18]. A Spatial Detrend correction was applied using the Agilent Feature Extraction algorithm. The following features and/or genes which did not conform to the established quality criteria were filtered: (a) non-uniform pixel distributed outliers and population replicate outliers according to the default Agilent feature extraction criteria, (b) spots not differentiated from the background signal (as estimated for each spot), (c) spots in the range of the negative controls.

To identify the genes specifically expressed in the CTC population of mCRPC patients, we considered signals obtained in the healthy controls as the background from non-specifically isolated blood cells, mainly lymphocytes. After discarding the genes expressed in healthy samples, the list of genes uniquely expressed in CTCs was composed by those genes present in at least 5 patients. The gene set characterizing CTC population was analyzed with the Ingenuity Pathway Analysis software version 20.0 (IPA, Qiagen, Redwood City, CA, USA) for network generation and the main signaling pathways involved.

### 2.4. CTCs Markers Validation by qRT-PCR

The CTCs were isolated from a larger cohort of patients, and the total RNA was extracted with the QIAmp viral RNA mini kit (Qiagen, Valencia, CA, USA) as previously stated (Leon Mateos et al., Oncotarget, 2017). Complementary DNA (cDNA) was obtained using SuperScript III reverse transcriptase (Invitrogen, Carlsbad, CA, USA), adding 11 μL of total RNA per reaction. Because of the very low cellularity of the samples, the cDNA was subjected to 14x pre-amplification cycles using TaqMan^®^ PreAmp Master Mix Kit (Applied Biosystems, Foster City, CA, USA) to increase the sensibility and stability. Finally, the pre-amplified 1:10 diluted cDNA was subjected to TaqMan RT-qPCR for selected genes (Supplementary Table S2). The mean threshold cycle (depicted as 40-Ct) for every candidate gene was normalized to *CD45*, a lymphocyte-specific marker that allows the quantification of non-specific isolation of blood cells [14,15,16]. The samples were run in duplicate and all the plates included negative controls.

### 2.5. Statistical Analysis

The statistical analyses, apart from the gene expression approach, were carried out using the software SPSS 22 for Macintosh (IBM Software Group, Chicago, IL, USA) and GraphPad Prism 7.0 for Windows (GraphPad Software Inc., San Diego, CA, USA). A validation analysis was performed using a two-tailed Mann–Whitney U-test, and the p-values for each marker were adjusted by the false discovery rate (FDR) test. Survival analyses were carried out by means of Kaplan–Meier and Cox regression analyses. For the survival analyses, the levels of CTC markers were grouped as high/low according to the median or percentile 70 value. Overall (OS) and progression-free (PFS) survival were calculated as the time between blood sample collection and patients’ progression/death or last disease control. For the correlation analyses, continuous variables were evaluated using the Pearson correlation coefficient (two-sided). For all the analyses, a probability lower than 5% was accepted as significant (*p* < 0.05).

## 3. Results

### 3.1. CTC Immunoisolation and Global Gene Expression Analysis

The strategy for CTC immunoisolation, RNA extraction, and amplification for hybridization onto cDNA microarrays was previously validated by our group [15,16]. Briefly, the CTCs were immunoisolated from 7.5 mL of peripheral blood from mCRPC patients (*n* = 9; Supplementary Table S1) at baseline. For that, we used magnetic beads coated with a monoclonal antibody towards the human Epithelial Cell Adhesion Molecule (EpCAM), a surface molecule highly expressed in carcinomas, especially in PC patients [19]. RNA from isolated CTCs was purified using a kit specifically designed for samples with low cellularity. In parallel, the same protocol was applied to blood samples from healthy donors (*n* = 6) to establish the baseline of background from unspecific non-CTC immunoisolation. Prior to the gene expression analysis, the presence of the isolated CTCs was confirmed by a CellSearch system quantification (Supplementary Table S1) or *KLK3* expression detection by RT-qPCR (data not shown).

In order to characterize the CTC population from the mCRPC patients after the immunoisolation, the purified RNA was amplified using the WTA2 whole transcriptome amplification method, and the complementary DNA was labeled and hybridized onto Agilent expression arrays (Gene Expression Omnibus, GEO. Accession number: GSE153514). After the initial pre-processing of the raw data, an average of 21,273 spots were filtered according to the criteria described in the Materials and Methods section, which represented 47.81 % of the spots in the microarray with a maximum of 28,867 (64.88%) and a minimum of 15,629 (35.12%). Normalization among all the microarray data was performed by the Quantile method implemented in the Limma package of the R statistical software. This method ensured that the A values (average intensities) had the same empirical distribution across microarrays, whilst leaving the M values (log-ratios) unchanged.

Then, we discarded the genes expressed in healthy samples and selected as CTCs characteristics those genes present in at least five patients at the baseline (Supplementary Table S3). This strategy led to the identification of a final set of 54 genes, 50 of them annotated genes, that were specific to the CTC population in our patients. It is important to remark on the presence in this list of *KLK3* (PSA) as the broadly accepted prostate cancer marker and *EpCAM*, the molecule used for the isolation of CTCs in our approach and the one classically used for CTC isolation in carcinomas. In addition, *BIRC5* was also found as a member of the list. This gene was previously described as a CTC marker from mCRPC patients by our group [14]. Of note, these results validate the analytical strategy to characterize the CTC population in mCRPC.

### 3.2. Biology of CTCs Isolated from mCRPC.

**Figure 1:**
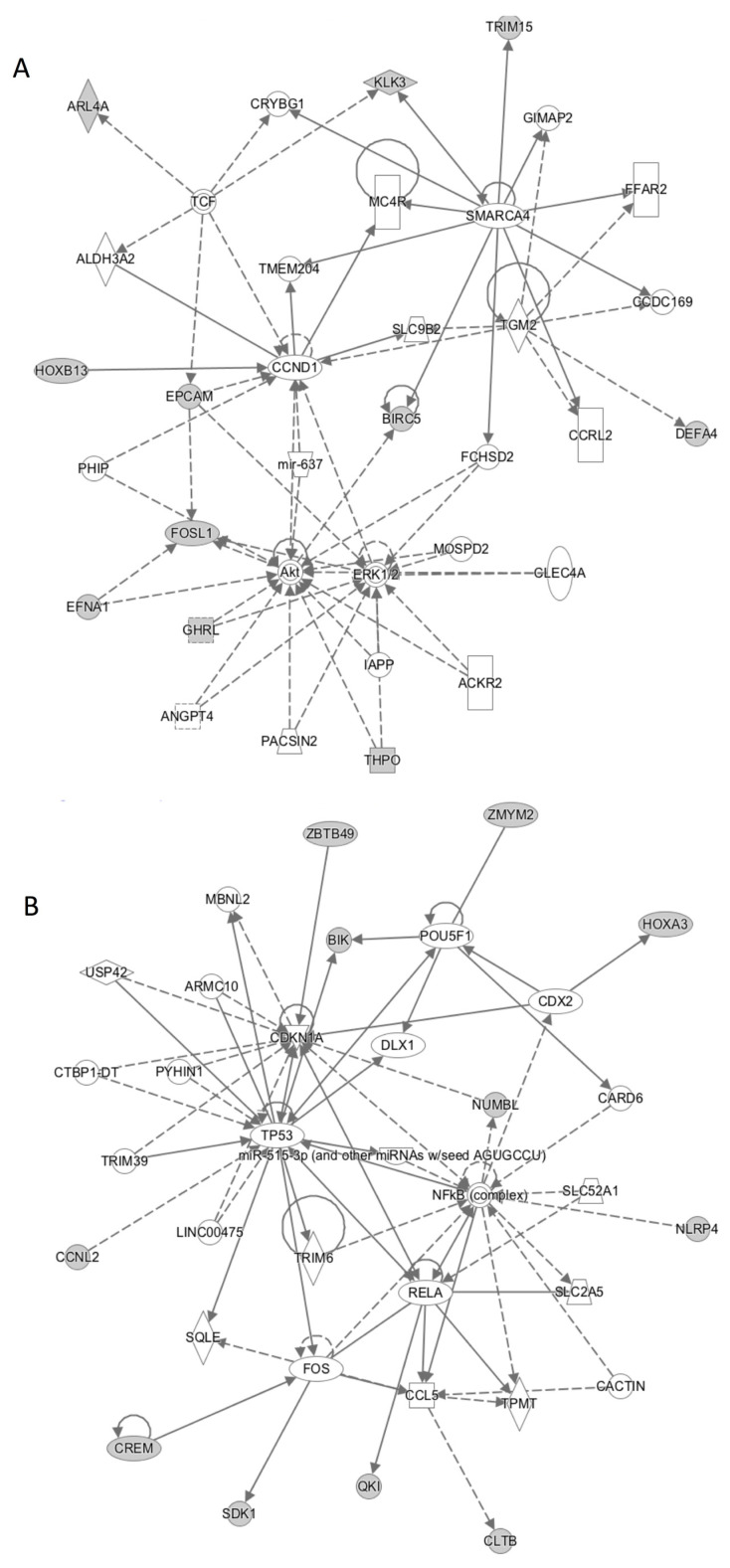
Two main molecular networks linked to the circulating tumor cell (CTC) profile identified after the global gene expression characterization. Network (**A**) has as central molecules *TP53* and *NFkB*, while network (**B**) relays on *AKT, ERK,* and *CCND1*.

The analysis of molecular pathways, gene networks, and biological functions associated with the list of genes specifically expressed in CTC immunoisolated from mCRPC was performed using an IPA tool. This analysis proposed a number of cellular functions and pathways related to the list of 50 CTC genes, with cancer as the disease most related to this gene signature, confirming the tumor origin of the isolated CTC population. We also found cell cycle, development, growth, and proliferation as important molecular and cellular functions involving these genes, illustrating relevant characteristics for a tumor cell (Supplementary Table S4). The main molecular networks linked to the CTC profile recognized *ERK, Akt, P53*, and *NFKB* as the central axis of the activity of the CTC profile identified (Figure 1). The IPA analysis also identified *CTNN1B* as an upstream regulator of these cellular functions in the subpopulation of CTC of mCRPC patients.

### 3.3. Validation of the CTC Gene Expression Profiling

**Figure 2:**
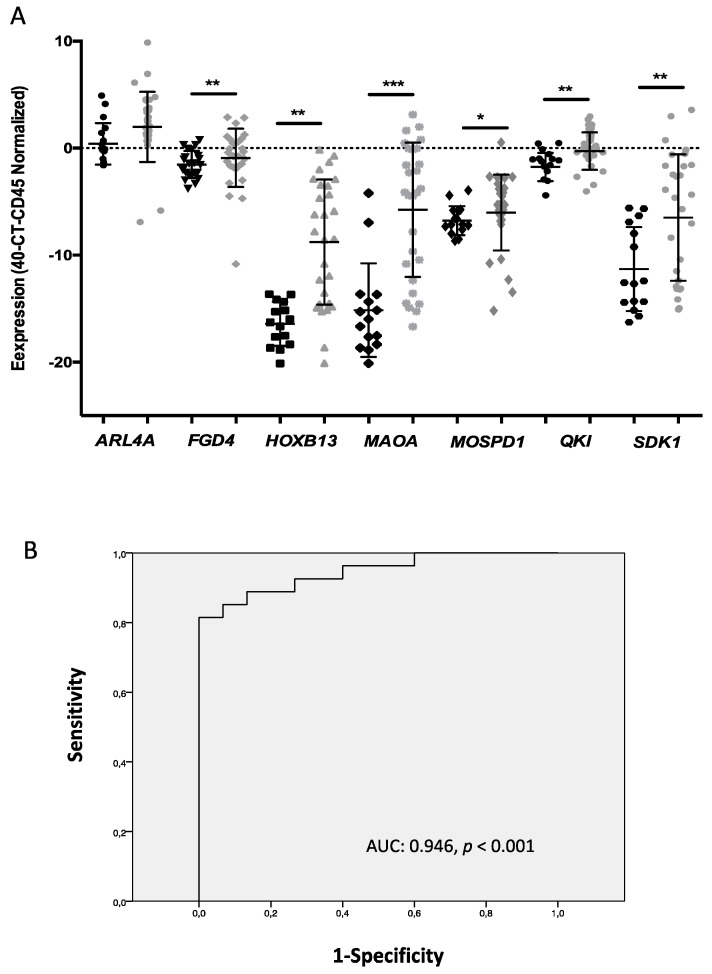
Validation of a CTC gene panel identified after a global gene expression array. (**A**) Gene expression levels of *ARL4, FGD4, HOXB13, MAOA, MOSPD1, QKI*, and *SDK1* analyzed in a cohort of 28 mCRPC patients and 15 controls. * *p* < 0.5, ** *p* < 0.05, *** *p* < 0.005 according to the Mann–Whitney U-test. (**B**) ROC curve analysis to discriminate the control and the patients’ group using a regression model combining the *FGD4, HOXB13, MAOA, MOSPD1, QKI*, and *SDK1* expression levels.

To further validate the results obtained after the global gene expression approach, we selected seven genes based on their high expression in the patients and their previous description in prostate cells and cancer. These genes were analyzed in a larger cohort of 28 mCRPC, which also includes the ones analyzed with the array. The panel of genes analyzed included *ARL4A*, *HOXB13*, *QKI*, *MAOA*, *MOSPD1*, *SDK1,* and *FGD4*. These genes mRNA levels were analyzed by q-RT-PCR in a fraction enriched with EpCAM-positive CTCs immunoisolated from whole blood samples before chemotherapy onset and compared with the expression found in a cohort of 15 healthy controls. As Figure 2A shows, the levels of *HOXB13*, *QKI*, *MAOA*, *MOSPD1*, *SDK1,* and *FGD4* were found to be statistically significantly increased in the patients in comparison with the controls. Besides, when the power to discriminate patients and controls was assessed by a receiver operated characteristics (ROC) curve, combining the expression of the markers, we obtained an area under the curve (AUC) = 0.946 (*p* < 0.001, CI: 0.88–1.00) (Figure 2B). Analyzed alone, the *MAOA* and *HOXB13* expression levels also showed a high power to detect the presence of disseminated disease (Supplementary Table S5). Importantly, these results validated the expression of the panel identified after the gene expression array on the EpCAM-positive CTC fraction isolated from the cohort of mCRPC patients.

Of note, there was no clear association between Gleason score, performance status, previous treatments, PSA, LDH and FA levels, and the CTCs panel (Supplementary Table S5). The impact of the CTC expression signature on the tumor evolution and the therapy response was also evaluated. For that, the expression of the markers was grouped as high/low taking into account the median or the percentile 70 value for each marker. After Kaplan–Meier analyses, a significantly longer PFS was observed in patients with a low expression of *MAOA, MOSPD1, QKI*, and *SDK1*, suggesting that CTCs expressing theses markers could be more resistant to chemotherapy (Supplementary Table S5). Curiously, these markers did not show an impact on the OS, while high expression levels of *HOXB13* were clearly associated with poorer OS rates (Table 2).

## 4. Discussion

Improvements in the knowledge of molecular determinants guiding PC have led to the approval of different new drugs to treat mCRPC patients. These treatments encompass androgen receptor-directed therapies (abiraterone, enzalutamide), immunotherapies, bone-targeting radiopharmaceuticals (radium-223), and cytotoxic chemotherapies (docetaxel, cabazitaxel) [3]. However, the development of personalized and sequential management strategies has been hindered by the impossibility of identifying distinct prognostic subgroups [20]. The interest in the CTC population, as the principal responsible for PC dissemination and a valuable source to characterize the tumor in real-time, has increased exponentially in recent years. In fact, changes in the CTC phenotype could reflect tumor evolution under the pressure of systemic therapies, providing a unique opportunity to gain insight into the mechanisms regulating prostate cancer biology [21,22,23].

In a previous work, we explored the expression of PC-associated genes in the CTC population from mCRPC patients and identified new CTCs markers of clinical interest to predict the patients’ outcomes [14]. Here, we performed a global gene expression approach to further characterize the CTC population from these patients with the goal to find the main actors behind PC aggressiveness. For that, we combined CTC immunoisolation based on EpCAM expression, accurate RNA extraction from a very low number of cells, whole-genome amplification, and a massive gene-expression profiling for the characterization of the biology of CTC, as we previously described for colorectal and lung cancer [15,16]. This profiling approach allowed us to identify 50 genes by subtracting the background of the non-specific isolation of blood cells obtained in a group of healthy controls, following the same procedure as patients. Validating our analytical strategy, we found PSA (*KLK3*) and EpCAM to be components of the CTC profile. Both genes are well accepted as specific markers of PC CTCs but also as molecules implicated in this tumor behavior [24,25,26,27]. In addition, the expression of the main part of the CTC genes was previously described in prostate tissues and most of them are implicated in key steps of prostate carcinogenesis, such as *PAGE2B* (Prostate-Associated Gene 2B Protein) [28], *MAOA* (Monoamine Oxidase A) [29], and *HOXB13* (Homeobox B13) [30]. Within this list, we also found *EFNA1*, which encodes a member of the Ephrin family of membrane receptors involved in cell migration, attachment and spreading, which has been also described as a potential marker of progression in PC [31]. *BIRC5,* known as Survivin, was also part of the CTCs signature, which has been strongly associated with PCa development, progression, and drug resistance [32,33,34] and previously identified in CTCs from mCRPC by our group [14].

From a global point of view, the genes characterizing the CTC population in the cohort of patients were associated with relevant functions for a tumor cell such as cell cycle, development, growth, and proliferation [35]. These functions are consistent with a subpopulation of tumor cells that must acquire an aggressive and invasive phenotype allowing dissociation from the primary tumor, the invasion of neighboring tissues, and their intravasation and survival in the blood flow [36]. For example, the activity of BIRC5 in CTCs could be important to prevent the mechanisms of cellular death induced by a high hostile environment such blood, since this molecule has a key role to inhibit apoptosis. In fact, it is known that regulation of apoptosis has a central role in the development of PC and its progression to an androgen-independent state, which is due, in part, to up-regulation of antiapoptotic genes such as Survivin [37,38]. Interestingly, our CTC profiling at baseline pointed out as relevant cell signaling pathways for CTC biology, such as *ERK*, *AKT*, *P53,* and *NFKB*, all of them classical tumor driver pathways in cancer [39,40,41].

With the aim of corroborating the results obtained after the gene expression array, seven genes (*ARL4A*, *HOXB13*, *QKI*, *MAOA*, *MOSPD1*, *SDK1,* and *FGD4)* were selected for validation in a larger cohort of mCRPC and healthy controls. These genes were prioritized based on their high expression in the arrays and their previous description in relation with the prostate and carcinogenesis. After their analysis by RT-qPCR, we found six of these genes (*HOXB13*, *QKI*, *MAOA*, *MOSPD1*, *SDK1,* and *FGD4)* characterizing the CTCs population from patients. Of note, *HOXB13* has an important role in the development of the separate lobes of the prostate gland, seminal vesicles, and epididymis [42]. The alteration of this gene has been previously implicated in the PC development and is known for regulating AR transcriptional activity during prostate tumorigenesis [43,44]. Of note, the *HOXB13* overexpression in CTCs from mCRPC was previously described by Miyamoto and collaborators and is associated with a more aggressive CRPC in terms of worse overall survival rates [45].

MAOA (Monoamine oxidase A) is a mitochondrial membrane enzyme that catalyzes the oxidative deamination of serotonin, melatonin, catecholamines, and other biogenic amines [46]. Several studies have reported high levels of *MAOA* transcripts in malignant prostate epithelium and associated them with higher Gleason grades and tumor severity [29,47]. On the other hand, the *QKI* gene codifies for Quaking protein, which has important signal transduction and RNA activation functions. In PC, positive AR expression often co-exists with higher *QKI* expression levels [48]. The expression of *FGD4* is also upregulated in PC and is associated with the increased aggressiveness of the disease. In fact, the inhibition of *FGD4* expression has been demonstrated to improve drug sensitivity of prostate cancer cells [49]. *MOSPD1* belongs to the transmembrane MSP (major sperm protein)-containing protein family and is thought to be implicated in proliferation and differentiation processes; however, it was not previously related to PC [50]. *SDK1* (Sidekick cell adhesion molecule 1) is regulated by androgen through the androgen-responsive serum response factor (SRF) [51], and its fusion with the α-metilacil-CoA racemasa (AMACR) was described in a high percentage of Chinese PC patients [52].

Interestingly, IPA analyses associated this heterogeneous gene panel with the regulation of MYC (Supplementary Figure S1). Activation of MYC is one of the most frequent genetic events linked to the promotion of androgen-independent growth of prostate cancer cells [53]. In fact, MYC amplifications are often found in tumor tissues from CRPC patients and this amplification is more evident after anti-androgen therapy [54,55]. Therefore, the CTCs signature identify in the study is clearly compatible with prostate tumor cells which developed mechanisms of resistance to the androgen regulation and the promotion of tumor progression. In this line, the high expression of *MAOA, MOSPD1, QKI,* and *SDK1* was associated with shorter progression-free survival rates in response to Docetaxel treatment, suggesting the existence of more aggressive disease.

## 5. Conclusions

Overall, the present study described a specific molecular profile of CTC isolated from mCRPC within Docetaxel treatment. This global gene expression analysis allowed us to get a better picture of the relevant actors in PC progression after androgen deprivation. We found a general stress survival phenotype in the CTC population of mCRPC patients partially based on cell proliferation and differentiation. Importantly, we identified a novel CTC signature, which could represent a valuable tool for CTC detection and as a prognostic and monitoring biomarker. Although a deeper validation of our results should be attempted in a bigger independent cohort of mCRPC, our results reinforced the value of CTC characterization as an alternative liquid biopsy that could be useful to improve the clinical management of these patients.

## Figures and Tables

**Table 1:** Clinical parameters of the metastatic castration-resistant prostate cancers (mCRPC) cohort.

Age (Years)	Mean (Range)
	69.6 (52–80)
Previous PT * surgery	***n* (%)**
yes	6 (21.4)
no	22 (78.6)
ECOG, PS **	
0	7 (25)
1	18 (64.3)
2	3 (10.7)
**Gleason score**	
≤7	15 (53.6)
>7	10 (35.7)
Unknown	3 (10.7)
**Metastasis location**	
Bone only	15 (53.6)
Lymph node + Bone	10 (35.7)
Any + Lung	3 (10.7)
PSA*** (ng/dL)	
Mean, range	445 (2–3238)
Median, range	136 (2–3238)
AP ****, (UI/L)	
Mean, range	617 (77–3115)
Median, range	461 (77–3115)
LDH ***** (UI/L)	
Mean, range	503 (121–1136)
Median, range	454 (121–1136)

* PT primary tumour; ** PS, performance status; *** PSA, prostate-specific antigen; **** AP, Alkaline phosphatase; ***** LDH, Lactate dehydrogenase.

**Table 2:** Kaplan–Meier analysis for CTC markers.

	Overall Survival (OS)		Progression Free Survival (PFS)	
Marker	Mean (95% CI)	*p*-Value	Mean (95% CI)	*p*-Value
*HOXB13*				
low	31.77 (22.76-40.79)	0.014 *	8.20 (6.20–10.20)	0.344
high	16.39 (8.86–23.92)		6.68 (4.18–9.18)	
*MAOA*				
low	30.66 (18.97–42.35)	0.338	8.73 (5.42–12.05)	0.028 *
high	23.51 (15.55–31.47)		6.47 (4.42–8.51)	
*FGD4*				
low	28.65 (18.49–38.81)	0.725	8.74 (6.63–10.85)	0.068
high	24.77 (15.55–34.00)		6.06 (4.00–8.12)	
*MOSPD1*				
low	30.01 (20.95–39.07)	0.264	8.74 (6.74–10.74)	0.006 *
high	20.57 (10.15–30.98)		5.39 (3.75–7.04)	
*QKI*				
low	29.27 (20.17–38.37)	0.507	8.78 (6.87–10.70)	0.022 *
high	22.32 (11.38–33.27)		5.34 (3.11–7.57)	
*SDK1*				
low	28.59 (19.64–37.53)	0.546	8.89 (7.07–10.56)	0.014 *
high	23.28 (12.21–34.35)		4.70 (2.15–7.23)	

* p ≤ 0.05 according to Log-Rank test; HR: hazard ratio; CI: confidence interval. Cut-off values were −3.8, −4.1, 0.4, −4.0, 0.5 and −2.0 for *HOXB13, MAOA, FGD4, MOSPD1, QKI* and *SDK1*, respectively.

## Supplementary Materials

The following are available online at https://www.mdpi.com/2077-0383/9/7/2066/s1: Figure S1: Main molecular network linked to the CTC panel analyzed by RT-qPCR. MYC was in center of the network. Table S1: Clinical characteristics of the 9 mCRPC included in the global gene expression array. Table S2: Taqman assays employed for the RT-qPCR. Table S3: List of genes specifically expressed in CTCs from 9 mCRPC patients identified after the gene expression array. Table S4: Main functions associated with the list of genes characterizing the CTC population of mCRPC patients after IPA analyses. Table S5: Correlation between baseline characteristics and CTCs profile.
