# Supplementary material for: Global Gene Expression Characterization of Circulating Tumor Cells in Metastasic Castration-Resistant Prostate Cancer Patients
Source: J Clin Med. 2020 Jul 1;9(7):2066. doi: 10.3390/jcm9072066 (PMC7408664; doi:10.3390/jcm9072066)
Supplement: Supplementary file 1 [file jcm-09-02066-s001.zip › Supplementary Figure 1. .pdf]

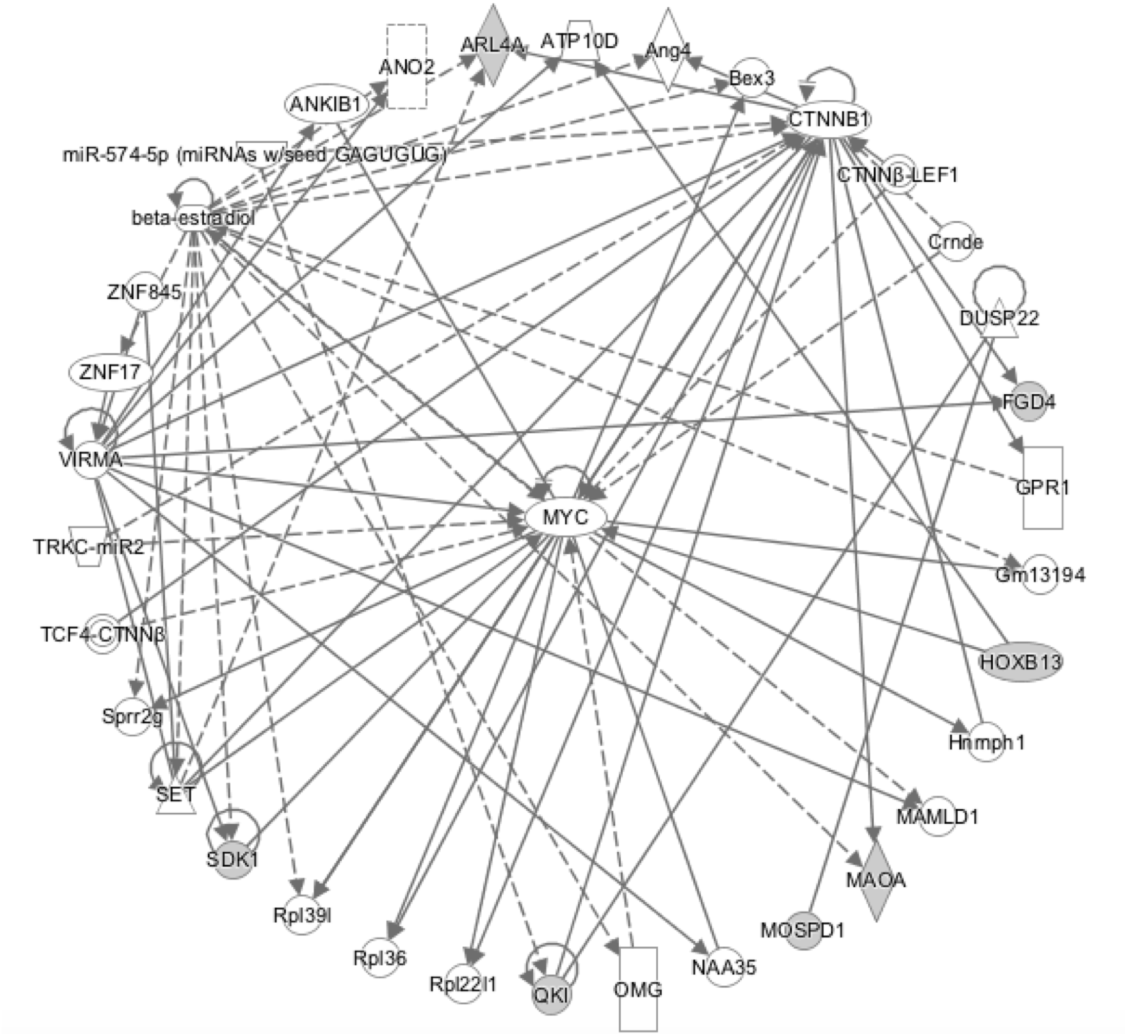

Supplementary Figure 1. Main molecular network linked to the CTC panel analyzed by RT-qPCR. Main regulation of these gene is centralized on MYC activity.
