# Supplementary material for: Global Gene Expression Characterization of Circulating Tumor Cells in Metastasic Castration-Resistant Prostate Cancer Patients
Source: J Clin Med. 2020 Jul 1;9(7):2066. doi: 10.3390/jcm9072066 (PMC7408664; doi:10.3390/jcm9072066)
Supplement: Supplementary file 1 [file jcm-09-02066-s001.zip › Supplementary Tables.docx]

| Code | **Age** | **ECOG** | **Gleason score**  **at diagnosis** | **Lymph node metastasis** | **Metastasis**  **site** | **Nº previous treatments** | **Baseline**  **PSA (ng/dl)** | **Number CTCs*** | **PFS (months)** |
| --- | --- | --- | --- | --- | --- | --- | --- | --- | --- |
| **Patient 2 11** | 64 | 1 | - | yes | Bone and lung | 3 | 196 | 55 | 7 |
| **Patient 5** | 80 | 1 | - | yes | Bone | 2 | 139 | 0 | 9 |
| **Patient 6** | 65 | 1 | - | yes | Bone | 2 | 1.929 | 59 | 6 |
| **Patient 9** | 52 | 1 | - | no | Bone | 3 | 409 | 78 | 4 |
| **Patient 11** | 73 | 0 | - | no | Bone | 3 | 233 | 12 | 10 |
| **Patient 13** | 79 | 1 | 9 | no | Bone | 4 | 715 | 12 | 6 |
| **Patient 17** | 71 | 1 | 8 | no | Bone | 2 | 3.115 | 199 | 9 |
| **Patient 21** | 68 | 1 | 9 | yes | Bone | 2 | 347 | 239 | 11 |
| **Patient 24** | 74 | 1 | 7 | yes | Bone | 1 | 279 | 21 | 6 |

**Supplementary Table 1.** Clinical characteristics of the 9 mCRPC included in the global gene expression array.

**Supplementary Table 2.** Taqman assays employed for the RT-qPCR.

| **Gene** | **TaqMan assay** |
| --- | --- |
| *PTPRC (CD45)* | Hs00894734_m1 |
| *HOXB13* | [Hs00197189_m1](https://www.thermofisher.com/taqman-gene-expression/product/Hs00197189_m1?CID=&ICID=&subtype=) |
| *MAOA* | [Hs00165140_m1](https://www.thermofisher.com/taqman-gene-expression/product/Hs00165140_m1?CID=&ICID=&subtype=) |
| *ARL4* | [Hs01932504_s1](https://www.thermofisher.com/taqman-gene-expression/product/Hs01932504_s1?CID=&ICID=&subtype=) |
| *FGD4* | [Hs01030780_m1](https://www.thermofisher.com/taqman-gene-expression/product/Hs01030780_m1?CID=&ICID=&subtype=) |
| *MOSPD1* | [Hs00219613_m1](https://www.thermofisher.com/taqman-gene-expression/product/Hs00219613_m1?CID=&ICID=&subtype=) |
| *QKI* | [Hs00916678_m1](https://www.thermofisher.com/taqman-gene-expression/product/Hs00916678_m1?CID=&ICID=&subtype=) |
| *SDK1* | [Hs01010140_m1](https://www.thermofisher.com/taqman-gene-expression/product/Hs01010140_m1?CID=&ICID=&subtype=) |

**Supplementary Table 3.** List of genes specifically expressed in CTCs from 9 mCRPC patients identified after the gene expression array.

| *GENE SYMBOL*  *TP53TG5*  *ENST00000372194*  *XM_001716578*  *THBS4*  *ROM1*  *MOSPD1*  *ARL4A*  *QKI*  *MAOA*  *BIRC5*  *FOSL1*  *PAGE2B*  *CREM*  *LOC100130700*  *A_33_P3215252*  ***KLK3***  *ZMYM2*  *ZBTB49*  *NGEF*  *TMTC1*  *HOXB13*  *HOXA3*  *TULP2*  *ENST00000409758*  *CADM4*  *ENST00000409646*  *EFNA1*  *BIK* | *GENE SYMBOL*  *HARBI1*  *TRIM15*  *WHAMML1*  *NKAIN4*  *ENST00000412934*  *C14orf72*  *AL050203*  *LEAP2*  *PPIL6*  *DEFA4*  *FGD4*  *ENST00000381747*  ***EPCAM***  *NUMBL*  *LOC286058*  *THPO*  *C9orf71*  *NBL1*  *CCDC19*  *SDK1*  *CCNL2*  *NLRP4*  *CLTB*  *C18orf25*  *TMCC2*  *GHRL* |
| --- | --- |

**Supplementary Table 4.** Main functions associated with the list of genes characterizing the CTC population of mCRPC patients after IPA analyses.

| **Molecules in the network** | **Score** | **Top functions** |
| --- | --- | --- |
| ACKR2, Akt, ALDH3A2, ANGPT4, ARL4A, BIRC5, CCDC169, CCND1, CCRL2, CLEC4A, CRYBG1, DEFA4, EFNA1, EPCAM, ERK1/2, FCHSD2, FFAR2, FOSL1, GHRL, GIMAP2, HOXB13, IAPP, KLK3, MC4R, mir637, MOSPD2, PACSIN2, PHIP, SLC9B2, SMARCA4, TCF, TGM2, THPO, TMEM204, TRIM15 | 22 | Cellular Growth and Proliferation, Connective Tissue Development and Function, Tissue Development |
| ARMC10, BIK, CACTIN, CARD6, CCL5, CCNL2, CDKN1A, CDX2, CLTB, CREM, CTBP1-DT, DLX1, FOS, HOXA3, LINC00475, MBNL2, miR-515-3p (and other miRNAs w/seed AGUGCCU), NFkB (complex), NLRP4, NUMBL, POU5F1, PYHIN1, QKI, RELA, SDK1, SLC2A5, SLC52A1, SQLE, TP53, TPMT, TRIM39, TRIM6, USP42, ZBTB49, ZMYM2 | 22 | Cell Cycle, Cellular Development, Cellular Growth and Proliferation |

**Supplementary Table 5.** Correlation between baseline characteristics and CTCs profile.

|  | ***HOXB13***  **(mean)** | ***MAOA***  **(mean)** | ***ARL4***  **(mean)** | ***FGD4***  **(mean)** | ***MOSPD1***  **(mean)** | ***QKI***  **(mean)** | ***SDK1***  **(mean)** |
| --- | --- | --- | --- | --- | --- | --- | --- |
| **ECOG** |  |  |  |  |  |  |  |
| 0 (n=7) | -12.4397 | -5.8801 | 2.0841 | 0.0971 | -4.7007 | 0.4113 | -7.9844 |
| 1-2 (n=21) | -7.5485 | -5.7213 | 1.9537 | -1.2609 | -6.4775 | -0.5055 | -5.9864 |
| **Gleason score at diagnosis** |  |  |  |  |  |  |  |
| >7 (n=11) | -9.0773 | -6.6863 | 1.8232 | -1.4539 | -6.1044 | -0.5670 | -7.5554 |
| ≤7 (n=14) | -8.3200 | -5.1727 | 2.8895 | 0.0082 | -6.5697 | 0.1633 | -4.8440 |
| **Prior hormone treatments** |  |  |  |  |  |  |  |
| 1-2 (n=14) | -6.5583 * | -5.4231 | 2.4769 | -1.1000 | -5,5539 | -,1317 | -5,6247 |
| > 2 (n=14) | -10.9844 | -6.0989 | 1.4957 | -0.7428 | -6,5128 | -,4209 | -7,3471 |
| **PSA at baseline**  **(ng/dl)** |  |  |  |  |  |  |  |
| <300 (n=21) | -8.9581 | -6.4077 | 2.0555 | -0.9752 | -6.3134 | -0.4308 | -6.9219 |
| ≥300 (n=7) | -8.2109 | -3.8210 | 1.7787 | -0.7599 | -5.1931 | 0.1871 | -5.1779 |
| **LDH baseline (UI/L)** |  |  |  |  |  |  |  |
| <411 (n=6) | -11.5457 | -10.9247* | 1.4370 | -0.6217 | -6.6417 | -0.1882 | -8.4593 |
| ≥411 (n=7) | -6.2251 | -3.8639 | 0.6679 | -0.1847 | -5.5033 | 0.1127 | -4.2346 |
| **PA baseline (UI/L)** |  |  |  |  |  |  |  |
| <225 (n=6) | -10.9027 | -9.4790 | 2.1310 | -2.6233 | -9.1682 * | -1.7557 | -9.1160 |
| ≥225 (n=19) | -8.3609 | -5.1302 | 1.7909 | -0.4806 | -5.3323 | 0.1279 | -5.3262 |
| *p<0.05 according to Mann Whitney U test. | | | | | | | |
